# New Computational Tools for *Brassica* Genome Research

**DOI:** 10.1002/cfg.394

**Published:** 2004-04

**Authors:** Christopher G. Love, Jacqueline Batley, Geraldine Lim, Andrew J. Robinson, David Savage, Daniel Singh, German C. Spangenberg, David Edwards

**Affiliations:** 1 Plant Biotechnology Centre Primary Industries Research Victoria Department of Primary Industries La Trobe University Bundoora Victoria 3086 Australia; 2 Victorian Bioinformatics Consortium Plant Biotechnology Centre La Trobe University Bundoora Victoria 3086 Australia

## Abstract

With the increasing quantities of *Brassica* genomic data being entered into the public domain and in preparation for the complete *Brassica* genome sequencing effort, there is a growing requirement for the structuring and detailed bioinformatic analysis of *Brassica* genomic information within a user-friendly database. At the Plant Biotechnology Centre, Melbourne, Australia, we have developed a series of tools and computational pipelines to assist in the processing and structuring of genomic data, to aid its application to agricultural biotechnology research. These tools include a sequence database, ASTRA, a sequence processing pipeline incorporating annotation against GenBank, SwissProt and *Arabidopsis* Gene Ontology (GO) data and tools for molecular marker discovery and comparative genome analysis. All sequences are mined for simple sequence repeat (SSR) molecular markers using ‘SSR primer’ and mapped onto the complete *Arabidopsis thaliana* genome by sequence comparison. The database may be queried using a text-based search of sequence annotation or GO terms, BLAST comparison against resident sequences, or by the position of candidate orthologues within the *Arabidopsis* genome. Tools have also been developed and applied to the discovery of single nucleotide polymorphism (SNP) molecular markers and the *in silico* mapping of *Brassica* BAC end sequences onto the *Arabidopsis* genome. Planned extensions to this resource include the integration of gene expression data and the development of an EnsEMBL-based genome viewer.


**Comparative and Functional Genomics**
*Comp Funct Genom* 2004; **5**: 276–280.
Published online in Wiley InterScience (www.interscience.wiley.com). **DOI:** 10.1002/cfg.394

**Conference Review**

### New computational tools for *Brassica* genome research

Christopher G. Love[1,2], Jacqueline Batley[1], Geraldine Lim[1,2], Andrew J. Robinson[1,2], David Savage[1,2], Daniel Singh[1,2], German C. Spangenberg[1,2] and David Edwards[1,2]*

[1] *Plant Biotechnology Centre, Primary Industries Research Victoria, Department of Primary Industries, La Trobe University, Bundoora, Victoria 3086, Australia*
[2] *Victorian Bioinformatics Consortium, Plant Biotechnology Centre, La Trobe University, Bundoora, Victoria 3086, Australia*

**Correspondence to:*
*David Edwards, Plant Biotechnology Centre, Primary Industries Research Victoria, Dept. of Primary Industries, La Trobe University, Bundoora, Victoria 3086, Australia.*
*E-mail: Dave.Edwards@dpi.vic.gov.au*

Received: 26 January 2004
Accepted: 6 February 2004

### Abstract

**With the increasing quantities of *Brassica* genomic data being entered into the public domain and in preparation for the complete *Brassica* genome sequencing effort, there is a growing requirement for the structuring and detailed bioinformatic analysis of *Brassica* genomic information within a user-friendly database. At the Plant Biotechnology Centre, Melbourne, Australia, we have developed a series of tools and computational pipelines to assist in the processing and structuring of genomic data, to aid its application to agricultural biotechnology research. These tools include a sequence database, ASTRA, a sequence processing pipeline incorporating annotation against GenBank, SwissProt and *Arabidopsis* Gene Ontology (GO) data and tools for molecular marker discovery and comparative genome analysis. All sequences are mined for simple sequence repeat (SSR) molecular markers using 'SSR primer' and mapped onto the complete *Arabidopsis thaliana* genome by sequence comparison. The database may be queried using a text-based search of sequence annotation or GO terms, BLAST comparison against resident sequences, or by the position of candidate orthologues within the *Arabidopsis* genome. Tools have also been developed and applied to the discovery of single nucleotide polymorphism (SNP) molecular markers and the *in silico* mapping of *Brassica* BAC end sequences onto the *Arabidopsis* genome. Planned extensions to this resource include the integration of gene expression data and the development of an EnsEMBL-based genome viewer. Copyright © 2004 John Wiley & Sons, Ltd.**

**Keywords:** **ASTRA; EnsEMBL; molecular marker; Gene Ontology (GO); bacterial artificial chromosome (BAC); genome sequencing**

### Towards a community *Brassica* database

Since the development of the Sanger sequencing method, the number of DNA sequences deposited in GenBank has continued to increase at an exponential rate. Recently, these sequences include 31 011 *Brassica* EST (expressed sequence tag) sequences produced by the Genoplante consortium in June 2003 and over 300 000 *Brassica oleracea* genomic sequences from TIGR, produced as part of their *B. oleracea* shotgun sequencing programme (**http://www.tigr.org/tdb/e2k1/bog1/**). There are further plans to sequence and release 30 000 *B. oleracea* cDNAs as a joint project between Agriculture and Agri-Food Canada (AAFC) and Horticulture Research International (HRI), UK (Graham King, Guy Barker and Carol Ryder, personal communication). Several research groups in industry and academia are also required to maintain confidential sequence data for several years before data can be released into the public domain, and as this data is released, the public *Brassica* sequence resource is expected to expand further. This growth in *Brassica* sequence data has led to

Copyright © 2004 John Wiley & Sons, Ltd.

the requirement for tools for the structuring and interrogation of this information. There are already several functional *Brassica* sequence databases developed by the *Brassica* community. These are generally based on different underlying database schemata and platforms, with their associated strengths and weaknesses. This complicates the integration and linking of the data in the different databases and the development of cross-platform tools. The increasing volume of *Brassica* sequence data and the start of the *Brassica rapa* genome sequencing programme provides the opportunity to develop a public community database, which can be distributed and managed by the *Brassica* bioinformatics research community, using a platform which is readily searchable and navigable by *Brassica* researchers. Recent discussions with the international *Brassica* research community and within the Multinational *Brassica* Genome Project steering committee suggests a pathway for a community *Brassica* database developed around open source software, MySQL and BioPERL.

### The ASTRA *Brassica* sequence database

At the Plant Biotechnology Centre, we have developed a *Brassica* ASTRA database for open distribution among the *Brassica* research community. The ASTRA annotation pipeline is a modular series of PERL scripts, which act as wrappers for sequence processing, annotation and database management. The flexibility and modular design of the ASTRA system enables the incorporation and expansion of data analysis and annotation modules, while the use of MySQL permits broad data integration and application on a scalable platform with limited initial cost outlay. The *Brassica* ASTRA database currently incorporates modules for sequence, protein and Gene Ontology annotation, comparative genome analysis with *Arabidopsis*, SSR discovery and PCR primer design (Figure 1). Further modules to incorporate genetic mapping data, gene expression data and a genome viewer are under development. All modules of the database can be accessed at: **http://hornbill.cspp.latrobe.edu.au/**

### Sequence processing and assembly

Where trace files are available, they are batch-processed using *phred* and *crossmatch* [5] to call and quality-score each base and screen for the removal of vector sequence contamination. All sequences are stored within a MySQL database in FASTA format. On addition of new data, sequences are assembled using TGICL [9]. Cluster ID, cluster members and assembled sequence alignments are then parsed to the MySQL database.

### Sequence annotation

Sequences within the database are annotated by comparison to the DNA and protein databases GenBank and SwissProt using BLAST [2]. The FASTA headers for the 10 most significant BLAST matches are parsed to the MySQL database along with HTML format files for each sequence alignment. HTML NCBI web-links are maintained, enabling direct, remote access to NCBI sequence annotation. The application of Gene Ontology (GO) terms for sequence annotation has greatly assisted the structured interrogation of sequence databases [13]. However, the manual annotation of sequences with GO terms is laborious and expensive. We have applied knowledge from the *Arabidopsis* research community [6] to assist in the transfer of *Arabidopsis* GO annotation to closely related *Brassica* EST sequences. We have compared each of the ASTRA *Brassica* sequences against a database of GO-annotated *Arabidopsis* sequences using BLAST. Where significant sequence similarity was identified, the *Arabidopsis* GO annotation was assigned to the *Brassica* sequence within the MySQL database. Users may query the annotation using a GO term key word search. The GO terms are also structured as a hierarchical tree and annotated *Brassica* sequences may be retrieved at branch points representing each hierarchical GO term. The practical application of GO annotation searches leads to the identification of candidate genes of interest with greater precision than SwissProt or GenBank annotation queries, due to the more precise structuring of GO annotation.

### SSR and SNP molecular marker discovery

Simple sequence repeats (SSRs) are important molecular marker tools for applied *Brassica* research. Traditionally, SSRs have been discovered through the laborious task of constructing and sequencing SSR-enriched genomic libraries. With the large volume of *Brassica* EST and genomic

Copyright © 2004 John Wiley & Sons, Ltd. 

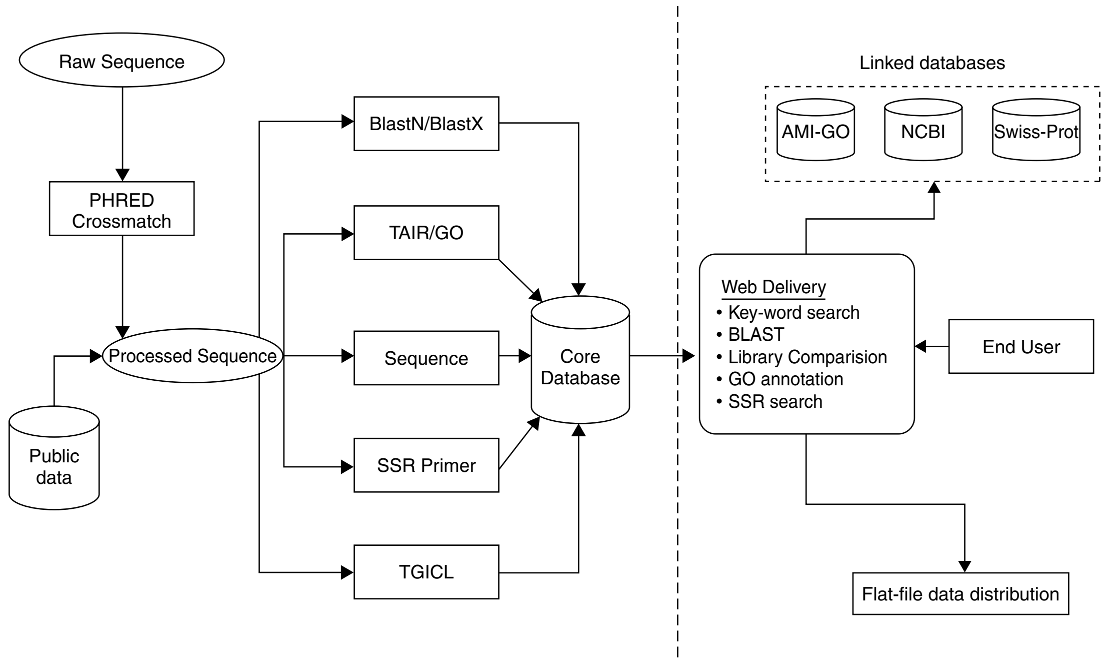

**Figure 1.** Schema for the *Brassica* ASTRA sequence processing and annotation database sequence data now available in the public domain, specific SSR discovery sequencing is no longer required, as SSRs may be identified within existing public sequence data. To assist the *in silico* discovery of SSRs, we have developed a bioinformatics tool that integrates SPUTNIK, an SSR finder [1], with Primer3, a PCR primer design programme [11], into one pipeline, SSR Primer [10]. On submission of one or more multiple FASTA-formatted sequences, the script screens each sequence for SSRs using SPUTNIK. Results are parsed to Primer3 for locus specific primer design. The script makes use of a web-based interface enabling remote use (**http://hornbill.cspp.latrobe.edu.au/**). A FASTA file of 300 870 *B. oleracea* genomic sequences (192 MB) derived from the TIGR *B. oleracea* shotgun sequencing programme was processed to identify PCR primer pairs for the amplification of 46 949 SSRs (18 194 dinucleotide, 14 096 trinucleotide, 6252 tetranucleotide and 8407 pentanucleotide). These SSR-containing genomic sequences and associated PCR primer pairs may also be accessed at: **http://hornbill.cspp.latrobe.edu.au**. All repeat types, including perfect, interrupted and compound, are detected and the minimum length of the SSR repeat, as well as PCR primer characteristics, can be stipulated by the user. Analysis of datasets representing ESTs from *B. napus, B. rapa* and *B. oleracea* maintained within the *Brassica* ASTRA database identified a total of 6625 SSR PCR primer pairs, representing 1273 dinucleotide, 4549 trinucleotide, 443 tetranucleotide and 359 pentanucleotide repeats.

To assist in the application of these SSRs to the comparative mapping of *Arabidopsis* and *Brassica* species, each of the *Brassica* EST sequences for which SSR primer pairs have been designed have been compared to the five completely sequenced *Arabidopsis* chromosomes [12] using BLAST [2]. Where significant sequence similarity ($e < 10^{-5}$) is identified, the *Arabidopsis* sequence location is parsed to the MySQL database and associated with the relevant *Brassica* sequence. This *in silico* SSR mapping data may be queried through a web-based form by entering a specific *Arabidopsis* chromosomal region to identify orthologous *Brassica* EST SSRs, along with the PCR primer pairs required for SSR amplification in *Brassica*. These EST SSR loci may be used for candidate gene based marker analysis, genetic mapping (targeting SSRs on different linkage groups) or diversity analysis. A recent modification to this SSR discovery

Copyright © 2004 John Wiley & Sons, Ltd. 

method seeks to determine the degree of polymorphism at specific EST SSR loci between different *Brassica* species or varieties and hence enable the *in silico* enrichment of polymorphic SSRs, saving the expense of PCR primer design to monomorphic SSR loci. The method requires the assembly of bulk sequence data followed by screening of consensus sequences for the presence of SSRs. Where an SSR is identified, SSR length is measured for each of the individual members of the assembly and used to calculate a polymorphism index for that locus. This will permit the selection of SSRs with predicted polymorphic value between several varieties or of an SSR length polymorphism between specified varieties of *Brassica*.

Single nucleotide polymorphisms (SNPs) and indels (insertions/deletions) are another highly valuable molecular marker for genetic analysis. They are used routinely in agriculture as ultra-high-throughput markers in crop breeding programmes. SNPs are valuable for genome mapping, offering the potential for generating high-density genetic maps, and have been applied to genetic diversity analysis for the understanding of genome evolution. SNPs within ESTs may also be used as perfect markers, when identified within candidate genes.

We have developed a computational method to identify candidate SNPs and small indels from EST data [3]. This method has been previously demonstrated to differentiate between true sequence polymorphisms and sequence error in maize [4], and we have recently applied this method to identify SNPs within public *Brassica* EST sequence data. This software initially uses TGICL to cluster and align EST sequences [9]. Using a redundancy-based approach, valid SNPs are distinguished from erroneous sequence by virtue of being represented multiple times in an alignment of sequence reads. For each candidate SNP, two measures of confidence are calculated, the redundancy of the polymorphism at a SNP locus and the co-segregation of the candidate SNP with other SNPs in the alignment. Application of this script to SNP discovery within 29 518 public *Brassica* ESTs identified 5414 candidate SNPs and 874 candidate indels. The complete data may be accessed at: **http://hornbill.cspp.latrobe.edu.au**. These publicly available SNP markers are of value to the *Brassica* community for marker-assisted selection (MAS), genetic mapping and candidate gene based marker studies. Further developments of this software will highlight potential non-synonymous base changes within coding regions and suggest whether the predicted amino acid change may alter protein structure and therefore function. Predicted SNP information may also suggest the presence of multigene families or indicate expression from the different genomes of allotetraploid *Brassica* species.

### Tools for sequencing the *Brassica* genome

This year sees the start of probably the most ambitious project yet for the long-term improvement of *Brassica* crops worldwide. Following in the footsteps of the *Arabidopsis* and rice researchers, the international *Brassica* research community has agreed to join forces, with the aim of sequencing one complete *Brassica* genome. In January 2003, the Multinational *Brassica* Genome Project steering committee, representing *Brassica* researchers from Australia, Canada, China, France, Germany, Korea, Poland, the UK and the USA, formulated a proposal to deduce the complete genome sequence of *Brassica rapa* by the end of 2007. Stage one of this genome project aims to sequence both ends from over 100 000 *Brassica* bacterial artificial chromosomes (BACs) from two libraries of *B. rapa* var. Chiifu by the end of 2004. These BAC end-sequences are to be used to identify seed BACs for initial sequencing and provide a framework for the identification of adjacent BACs for genome walking. This project creates many challenges and opportunities for *Brassica* bioinformatics as, unlike previous plant genome sequencing projects, sequencing the *Brassica* genome should be greatly assisted by the availability of the complete sequence of a closely related and syntenic plant, *Arabidopsis*.

The primary analysis of *Brassica* genome sequence data is performed using a BAC end-mapping tool developed as a collaborative project between Chungnam National University, Daejeon, Korea and the Plant Biotechnology Centre, Melbourne, Australia. This tool uses BLAST [2] to identify *Arabidopsis* genomic locations with significant sequence similarity to the sequenced ends of *B. rapa* BACs. Where sequences from both ends of a single BAC show similarity to *Arabidopsis* genomic sequence within a 500 000 bp region of the *Arabidopsis* genome and orientated

Copyright © 2004 John Wiley & Sons, Ltd. 

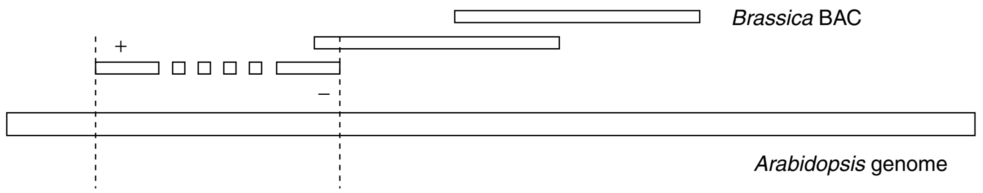

**Figure 2.** In silico mapping of *Brassica* BACs to the *Arabidopsis* genome in the opposite directions to each other, a co-linear, syntenic region of *Brassica/Arabidopsis* is indicated (see Figure 2). This enables a preliminary analysis of potential overlapping BACs syntenic to fully sequenced regions of the *Arabidopsis* genome and provides an initial validation of BAC contig assembly.

### Future directions

The BAC end-mapping tool is expected to be the first of a new era of coordinated *Brassica* community bioinformatic tool development. Work is under way to identify the most appropriate genome tools available, and establish where further coordinated tool development is required. Future work includes the development of an EnsEMBL-based *Brassica* genome database, in association with the *Arabidopsis* EnsEMBL database currently under construction at the University of Nottingham, UK [7]. This database will assist in the incorporation and integration of a variety of gene expression and genetic mapping data developed by the international *Brassica* community. The EnsEMBL database will be further complemented by the application of powerful scalable vector graphics (SVG) genome viewer technology developed by Christopher Lewis at Agriculture Canada [8]. By working together in a coordinated manner, the international *Brassica* research community now has the potential to capitalize on the advances in 'omics' technologies, make significant agronomic improvements to these important crops and bring *Brassica* research to the forefront of modern molecular science.

### References

1. Abajian C. 1994. SPUTNIK: **http://abajian.net/sputnik/**.
2. Altschul SF, Madden TL, Schäffer AA, *et al.* 1997. Gapped BLAST and PSI–BLAST: a new generation of protein database search programs. *Nucleic Acids Res* **25**: 3389–3402.
3. Barker G, Batley J, O'Sullivan H, *et al.* 2003. Redundancy based detection of sequence polymorphisms in expressed sequence tag data using AutoSNP. *Bioinformatics* **19**: 421–422.
4. Batley J, Barker G, O'Sullivan H, *et al.* 2003. Mining for single nucleotide polymorphisms and insertions/deletions in maize expressed sequence tag data. *Plant Physiol* **132**: 84–91.
5. Ewing B, Hillier L, Wendl MC, *et al.* 1998. Base-calling of automated sequencer traces using phred. I. Accuracy assessment. *Genome Res* **8**: 175–185.
6. Huala E, Dickerman A, Garcia-Hernandez M, *et al.* 2001. The *Arabidopsis* Information Resource (TAIR): a comprehensive database and web-based information retrieval, analysis, and visualization system for a model plant. *Nucleic Acids Res* **29**: 102–105.
7. James N, Craigon D, Gill G, *et al.* 2004. AtEnsembl — a new *Arabidopsis* genomic resource. Plant and Animal Genome XII Conference; 999: **http://www.intl-pag.org/12/abstracts/P99_PAG12_999.html**.
8. Lewis CT, Sharpe AG, Lydiate DJ, Parkin IAP. 2003. The *Brassica/Arabidopsis* comparative genome browser: a novel approach to genome browsing. *J Plant Biotechnol* **5**: 197–200.
9. Pertea G, Huang X, Liang F, *et al.* 2003. TIGR gene indices clustering tools (TGICL): a software system for fast clustering of large EST datasets. *Bioinformatics* **19**: 651–652.
10. Robinson AJ, Love CG, Batley B, *et al.* 2004. Simple sequence repeat marker loci discovery using SSR Primer. *Bioinformatics* (in press). DOI:10.1093/bioinformatics/bth104
11. Rozen S, Skaletsky HJ. 2000. Primer3 on the WWW for general users and for biologist programmers. In *Bioinformatics Methods and Protocols: Methods in Molecular Biology*, Krawetz S, Misener S. (eds). Humana: Totowa, NJ; 365–386.
12. The *Arabidopsis* Genome Initiative. 2000. Analysis of the genome sequence of the flowering plant *Arabidopsis*. *Nature* **408**: 796–815.
13. The Gene Ontology Consortium. 2000. Gene Ontology: a tool for the unification of biology. *Nature Genet* **25**: 25–29.

Copyright © 2004 John Wiley & Sons, Ltd. 
